# Immunomodulation From Moderate Exercise Promotes Control of Experimental Cutaneous Leishmaniasis

**DOI:** 10.3389/fcimb.2019.00115

**Published:** 2019-05-03

**Authors:** Rodrigo Terra, Pedro J. F. Alves, Ana K. C. Lima, Shayane M. R. Gomes, Luciana S. Rodrigues, Verônica P. Salerno, Silvia A. G. Da-Silva, Patricia M. L. Dutra

**Affiliations:** ^1^Discipline of Parasitology, Department of Microbiology, Immunology and Parasitology, State University of Rio de Janeiro, Rio de Janeiro, Brazil; ^2^Discipline of General Pathology, Department of Pathology and Laboratories, State University of Rio de Janeiro, Rio de Janeiro, Brazil; ^3^Laboratory of Exercise Biochemistry and Molecular Motors, School of Physical Education and Sports, Universidade Federal do Rio de Janeiro, Rio de Janeiro, Brazil

**Keywords:** exercise, leishmaniasis, immune response, Balb/c, control of infection

## Abstract

Physical exercise has been described as an important tool in the prevention and treatment of numerous diseases as it promotes a range of responses and adaptations in several biological systems, including the immune system. Studies on the effect of exercise on the immune system could play a critical role in improving public health. Current literature suggests that moderate intensity exercise can modulate the Th1/Th2 dichotomy directing the immune system to a Th1 cellular immune response, which favors the resolution of infections caused by intracellular microorganisms. Leishmaniasis is a group of diseases presenting a wide spectrum of clinical manifestations that range from self-limiting lesions to visceral injuries whose severity can lead to death. The etiological agents responsible for this group of diseases are protozoa of the genus Leishmania. Infections by the parasite *Leishmania major* in mice (Balb/c) provide a prototype model for the polarization of CD4+ T cell responses of both Th1 (resistance) or Th2 (susceptibility), which determines the progression of infections. The aim of this study was to evaluate the effect of exercise on the development of *L. major* experimental infections by scanning the pattern of immune response caused by exercise. Groups of Balb/c mice infected with *L. major* were divided into groups that preformed a physical exercise of swimming three times a week or were sedentary along with treatment or not with the reference drug, meglumine antimoniate. Animals in groups submitted to physical exercise did not appear to develop lesions and presented a significantly lower parasite load independent of drug treatment. They also showed a positive delayed hypersensitivity response to a specific *Leishmania* antigen compared to control animals. The IFN-γ/IL-4 and IFN-γ/IL10 ratios in trained animals were clearly tilted to a Th1 response in lymph node cells. These data suggest that moderate intensity exercise is able to modulate the Th1 response that provides a protective effect against the development of leishmanial lesions.

## Introduction

Regular physical exercise promotes a series of responses and physiological adaptations that are dependent on different aspects of the activity such as volume and intensity (American College of Sports Medicine Position Stand American Heart Association, 1998, 2007; Walsh et al., 2011b). The metabolic changes from exercise have been associated to alterations in immune function (Pedersen and Hoffman-Goetz, 2000), which can directly influence the immune response of a host to an infectious agent (Lili and Cheng, 2007). Intense exercise favors the resolution of bacterial infections by promoting a predominance to a Th2 type response (Malm, 2004, reviewed by Terra et al., 2012). Prolonged and strenuous exercise decreases the expression of Toll-Like Receptors in macrophages and compromises the presentation of antigens to T lymphocytes, which diminishes the Th1 inflammatory response. This anti-inflammatory effect prevents tissue damage caused by inflammatory mediators and reduces the risk of chronic inflammatory diseases, but increases the susceptibility to infections by intracellular microorganisms (Gleeson, 2006). Moderate exercise, in contrast, induces the immune system to a predominately Th1 type response that favors the resolution of viral infections and control of infections caused by intracellular microorganisms (Gleeson, 2006, 2007), which can include the bacteria *Mycobacterium tuberculosis* and *Listeria monocytogenis* along with the protozoa *Toxoplasma gondii, Trypanosoma cruzi*, and *Leishmania* spp. (Bogdan, 2008).

Parasites from the *Leishmania* genus are the etiological agents responsible for leishmaniasis, a vector-borne group of diseases that are endemic in 102 countries and territories distributed throughout Europe, Africa, Asia, and the Americas (Alvar et al., 2012; World Health Organization, 2018). Approximately 350 million people are at risk of infection with an annual incidence of around 1.6 million and a prevalence of 12 million individuals (World Health Organization, 2018). Phlebotomine sandflies are the principle vector of transmission and the macrophages of an infected person are the main host cell (Lainson et al., 1997). Based on the clinical manifestations and the parasites species involved, leishmaniasis can be present three main forms: visceral (also known as kala-azar, the most serious form that can be lethal), cutaneous (the most common), and mucosal (Stark et al., 2006; Shah et al., 2010; Badirzadeh et al., 2013; McCall et al., 2013; Organização Pan Amaricana De Saúde and, 2018). Cutaneous leishmaniasis (CL) is caused by several species of dermotropic *Leishmania*, such as *L. tropica* and *L. major* in the Old World along with *L. braziliensis* and *L. amazonesis* in the New World (Carvalho et al., 2005; Samy et al., 2014; Kahime et al., 2016). Mucosal leishmaniasis, also known as or mucocutaneous, is most commonly observed with infections by species restricted to South America, principally from the sub-genius *Viannia*, such as *L. braziliensis* and *L. panamensis* (Miranda Lessa et al., 2007). Visceral leishmaniasis is caused by viscerotropic species as *L. donovani* (Old World) and *L. infantum* (New World) (Momen et al., 1993).

Control of all the clinical forms of leishmaniasis appears to depend on type 1 immune response. Interferon-gamma (IFN-γ) and tumor necrosis factor (TNF) -α and -β, from the Th1 profile, are known to be involved in the resistance and elimination of the parasites, while Th2 cytokines such as IL-4 and IL-10 are linked to susceptibility to infections by *Leishmania* (Von Stebut, 2007; de Assis Souza et al., 2013). However, an exacerbation of the Th1 type response (hyperergia) leads to increased tissue destruction as observed in mucocutaneous leishmaniasis (Martin and Leibovich, 2005; Mendes et al., 2013).

The mouse strain Balb/c provides a laboratory model for the study of *Leishmania* infections, because this strain toward a Th2 (susceptibility) response, which determines the progression of infections (Fritzche et al., 2010; Barthelmann et al., 2011). The aim of the current study was to evaluate the effect of moderate exercise on the development of *L. major* experimental infections in Balb/c. Mice were infected with *L. major* and divided into a number of cohorts to analyse the impact of a swimming exercise (three times a week for 12 weeks) in combination or not with treatment with the reference drug, meglumine antimoniate (Glucantime®). In addition, the pattern of immune response caused by exercise was measured. The data obtained demonstrate that moderate intensity exercise was able to modulate the Th1 response, suggesting a protective effect of exercise on the development of leishmanial lesions.

## Materials and Methods

### Chemicals

Kits for measuring cytokines were purchased from R&D Systems (Minneapolis, MN, USA). Fetal calf serum (FCS) was purchased from Cultilab Co (Campinas, São Paulo, Brazil). DMEM culture medium, Schneider medium, bacterial lipopolysaccharide (LPS), concanavalin A (ConA), and all other chemicals used in this study were purchased from Sigma (St. Louis, MO, USA). The Glucantime® and the Ketamine were kindly provided by Oswaldo Cruz Institute and University Hospital Pedro Ernesto, respectively.

### Animals

A total of 94 male BALB/c mice (*Mus musculus*) were included in this study. Animals were housed in mini-isolators at 22–24°C on shelves ventilated by an IVC filter system (Model Domi AL20 system; Alesco®) with a 12 h light/dark cycle. Food and water was provided *ad libitum*. Animals were sacrificed using ketamine hydrochloride (7.5 mg), 48 h after the last training session. Popliteal lymph nodes and paws were immediately isolated following the sacrifice. This study was approved by the Ethics Committee for Experimental Use and Animal Care at the Biology Institute Roberto Alcântara Gomes (Protocol number CEA/043/2009). Animals were divided into eight cohorts (*N* = 8). In the case of lactate and MDA (lipid peroxidation) dosages, the animals were divided into three cohorts (*N* = 5). After 12 weeks of infection, the mice were scarified using CO_2_ chamber.

### Microorganisms

Promastigotes of the LV39 strain of *Leishmania major* were grown as previously described (Terra et al., 2013). Briefly, parasites were cultured in Schneider's medium supplemented with 2 mM glutamine, 100 units/mL penicillin, 100 mg/mL streptomycin and 20% fetal calf serum in a humidified incubator at 26°C. This strain was kindly provided by Dr. George dos Reis.

### Infection Model

Mice were infected in the plantar cushion with 2 × 10^6^ *L. major* promastigotes suspended in 20 μl of PBS. Lesion development was measured weekly using a caliper ruler (Mitutoyo, Brazil). The lesion size was calculated by the difference between the sizes of infected paw in relation to non-infected one.

### Groups

C—Control, E—Exercised—animals trained for 12 weeks, I—Infected—animals infected with 2 × 10^6^ *L. major* promastigotes, IE—Infected and exercised—infected animals trained for 12 weeks, from the first week of infection. IT—Infected with treatment—Animals infected and treated with therapeutic dose (8 mg) of Glucantime® after the onset of injury. ITE—Infected with treatment and exercised post-lesion—Animals infected, treated with therapeutic dose (8 mg) of Glucantime® and exercised after the onset of injury (from 6th week). IEP—Infected and exercised post-lesion—infected animals trained (for 6 weeks) after the onset of injury (from 6th week). EAPI -Trained during 6 weeks, infected and exercised—Animals trained for 6 weeks, infected and trained for 12 weeks.

### Treatment Protocol

Animals were treated with a therapeutic dose (8 mg @ ~500 mg/kg) of Glucantime® by an intraperitoneal application 5 times/week over a 12 week span. The treatment started after the onset of injury (6th week).

### Exercise Protocol and Physical Tests

Animals were subjected to a previously described swimming exercise protocol for 30 min X 3 times per week for 6 or 12 weeks with some modifications (Terra et al., 2013). The training started 6 weeks before the infection (EAPI), 48 h after the infection (IE) or after 6 weeks from the infection (ITE and IEP). Animals were introduced into a tank (50 × 50 × 40 cm) with a water depth of 30 cm and a temperature of 32 ± 2°C that stimulated swimming, herein referred to as the study protocol. To meet the desired exercise intensity, weight was attached to their tails. The initial mass was 2% of the measured body mass (BM) of each individual animal. The load was increased to 4% BM in week 4 and 6% BM in week 6. Animal BM was measured weekly.

Animals in the exercise groups (E, IE, ITE, IEP, and EAPI) were submitted to multiple physical capacity tests starting on the initial week and repeated on the fourth, 8th and 12th weeks. The other groups (C, I and IT) were submitted to physical capacity tests just in starting on the initial week and repeated on the 12th week. The test consisted of a timed swimming session with a 2% BM load until the point of fatigue, which was defined as the moment when the animal remained submerged for 10 s without returning to the surface for breath. An intense exercise test was also applied prior to measure lactate and MDA levels that consisted of a swimming session until the exhaustion with the addition of weight equal to 6% BM.

### Lactate

To verify that the study protocol reflected a protocol of moderate intensity, we measured the blood lactate in the animals after three different situations: rest, submitted to study protocol and submitted to a strenuous exercise protocol. The animals used in this evaluation (*N* = 15) did not participate of the experimental groups. Blood lactate levels were measured using a lactometer (Accusport®) from a whole blood sample (~10 μl) collected from a small incision in the tail. Measurement were performed on three groups: sedentary, moderate exercise and intense exercise, at rest and, for the exercise groups, immediately after an exercise session (acutely, before beginning training).

### Evaluation of Lipid Peroxidation Indices

The lipid peroxidation dosage (thiobarbituric acid reactive substances-TBARs) was performed as previously described (Keles et al., 2001). Three groups were used: rest, moderate exercise and intense exercise. The animals used in this dosage (*N* = 15) did not participate of the experimental groups. Immediately after the exercise session, animals were anesthetized with ketamine hydrochloride (7.5 mg) and blood was collected by a cardiac puncture into tubes with EDTA. The blood was centrifuged for 10 min at 1,000 g and the plasma was isolated to perform an assay for lipid peroxidation. The plasma (50 μL) was mixed with 200 μL of 10% TCA and 150 μL of potassium phosphate buffer (100 mM, pH 7.4) and incubated at room temperature for 10 min before centrifugation (2,000 × g for 15 min). The supernatant was collected and then, 500 μL thiobarbituric acid (0.67%) was added followed by an additional incubation at 95°C for 60 min. The samples were then cooled for 5 min and subjected to vortex. Finally, the absorbance was measured at 532 nm in a microplate reader, TP reader Thermo Plate. The MDA (malondialdehyde) concentrations were evaluated using a TMP (1,1,3,3-Tetramethoxypropane) standard curve.

### Antigen Parasite Preparation

After obtaining sufficient numbers of parasites in the first culture pass (P1) at the beginning of the stationary phase (determined by a growth curve), they were centrifuged at 1,300 g for 10 min and washed twice with PBS to remove serum. The pellet was resuspended in DMEM medium and the parasite number adjusted to 2 × 10^8^/mL. The promastigotes were submitted to three cycles of freezing-thawing in liquid nitrogen for complete lysis. The total antigen thus obtained was aliquoted and stored at −20°C until use. For the quantification of protein in the lysate, the Lowry method (Lowry et al., 1951) was used.

### Delayed Type Hypersensitivity (DTH)

DTH was evaluated using total antigen of *L. major* (1 μg/μl). The antigen (20 μl) was inoculated into the plantar cushion of non-infected paw. The extent of swelling was measured using a caliper rule (Mitutoyo, Brazil) 48 h after inoculation. The DTH was expressed as the difference between the paw sizes before and after the antigen inoculation.

### Lymph Node Cell Culture

The popliteal lymph nodes were harvested as previously described (Terra et al., 2013). Briefly, after the excision of the lymph nodes, a single cell suspension for each group was prepared by forcing tissue fragments through a stainless-steel mesh. The cell suspension (500 μL) was plated at 2 × 10^6^ cells/mL in 24-well plates in the absence or presence of the parasite antigen (10 μg/mL) and incubated at 37°C in atmosphere of 5% CO_2_ for 48 h. Supernatants were collected for cytokine measurements.

### Infected Paws

After sacrifice, infected paws were collected and weighed. After removal of claws and skin, a single cell suspension for each group was prepared by forcing tissue fragments through a stainless-steel mesh into Schneider medium. The product of maceration was separated by centrifugation (1,300 × g for 20 min). From the supernatant, measurements for cytokines levels were made and the parasite load determined.

### Measurement of Cytokine Production

The levels of the cytokines IFN-y, IL-4, IL-10, TNF, TGF-β, and IL-12 in the supernatants of cultured lymph node cells and isolated infected paws were measured by sandwich ELISA (R&D Systems, USA) utilizing a standard curve of recombinant murine cytokines and antibodies according to the manufacturer's instructions. Briefly, capture antibodies were plated into a 96-well ELISA plates and incubated at room temperature for 18 h (TNF-α = 0.8 μg/mL, other cytokines - 4 μg/mL), washed 3 times with PBS pH 7, 2, TWEEN 20 (0.05%) and blocked with PBS (pH 7.2) with 1% bovine serum albumin (BSA) for 1 h at room temperature. For TGF-β, the blocking solution also contained TWEEN 20 (5%) and sodium azide (0.05%) and that of IFN-γ, 0.05% sodium azide. After blocking, the plates were washed 3 times with wash buffer and the samples added (TGF-β dosing samples were previously activated by 1N HCl solution and neutralized by the addition of 1.2 N NaOH, 0.5 M HEPES). Then, the systems were incubated at room temperature for 2 h, washed three times with wash buffer and followed by the addition of biotinylated antibodies to cytokines TGF-β, TNF-α (200 ng/ml each), IL-10, IL-12p40 (400 ng/ml), IFN-γ (800 ng/ml), and IL-4 (600 ng/ml). Again, the samples were incubated for 2 h at room temperature and subsequently washed 3 times. The detection was achieved with Streptavidin HRP (conjugated to peroxidase and diluted 1:250). After washing, 0.1 M sodium citrate-acetate buffer, pH 4.0, containing 0.02% Tetramethylbenzidine (TMB), 0.02% hydrogen peroxide (H_2_O_2_) and 5% DMSO was added in the absence of light, for 20 min at room temperature. After this period, the reaction was stopped by adding 2N sulfuric acid (H_2_SO_4_) and read at 450 nm in a microplate reader, TP reader Thermo Plate.

### Parasite Load

To obtain an estimative from number of live parasites, these were quantified as described before, with modifications (Kalama and Nanda, 2009). Briefly, to obtain the parasites, after the sacrifice of the animals, the infected paws were cut, weighed, had claws, and skin removed and were macerated in Schneider's medium (8 mL), with samples of the eight animals per group grouped as a pool. The resultant supernatant was used for parasites quantification. A volume of this supernatant was distributed into a 96-well plate through a serial dilution of 1:10 to 1:10,000 using Schneider medium supplemented with 100 units/mL of penicillin, 100 mg/mL of streptomycin and 20% fetal calf serum. Plates were cultured at 26°C until the presence of parasites was observed in group I (7th day). Then, the parasites were quantified in Neubauer Chamber, and the number of parasites obtained was divided by the total weight (gram) of infected paws tissue.

### Statistical Analysis

Normality testing was performed for all samples. One-way ANOVA followed by the Tukey multiple comparisons test were used to analyze the statistical significance of cytokine concentrations from cultured lymph node cells and supernatant of infected paws. One-way ANOVA followed by the Dunnet test were used to analyze the statistical significance of DTH and parasite load. Differences were considered significant when *P* < 0.05.

## Results

### Control of Exercise Intensity Through Physical Capacity Tests, Blood Lactate Concentration, and Lipid Peroxidation

Animal physical performance was evaluated by a maximum physical capacity test that was executed every 4 weeks to verify the adaptation of each animals to the swimming exercise. Exercise intensity was adjusted by an increase in the weight overload (% BM) or time of the exercise to maintain a moderate level of physical exertion. By the 8th week of exercise, the IEP, EAPI groups did not adapt satisfactorily to the increase of the overload to 6% of BM. The weight overload of 4% BM was maintained with an increase in time, since this was significantly different from the previous test for the same overload (*P* < 0.05). There was no significant difference in the maximum exercise time for the IE and E groups as a function of the overload increase over the weeks. However, the exercised groups showed an improvement in swimming capacity when compared to control groups (Table 1). All trained groups showed a significant increase in swimming time (>100%) during the physical fitness test. This data indicated that the exercise protocol used here, the study protocol, was able to produce positive training effects.

**Table 1:** Maximal physical capacity of animal groups in a timed swim test at the start and end of the study.

**Group**	**Swim time (min)**		**Differences (%)**
	**Initial week**	**Final week**	
C	16.41 ± 4.45	22.02 ± 11.77	↑34
E	30.98 ± 11.31	62.29 ± 3.16^*^	↑101
I	41.82 ± 7.79	35.57 ± 9.07	↓15
IT	43.09 ± 5.80	40.00 ± 7.21	↓7
ITE	38.23 ± 6.95	69.73 ± 8.87^*^	↑82
IE	22.02 ± 11.78	59.37 ± 12.32^*^	↑170
IEP	23.28 ± 12.57	60.39 ± 11.77^*^	↑159
EAPI	40.23 ± 7.47	69.45 ± 6.29^*^	↑73

* *P < 0.05. The animals were submitted to maximal physical capacity test in the starting and at the final of experiment. In the case of E, IE, ITE, IEP, and EAPI groups, the test also was applied every 4 weeks from the initial week. The time was measured in minutes*.

^*^P < 0.05. Arrows indicate increase or decrease in exercise time in relation to the first week.

**Figure 1:**
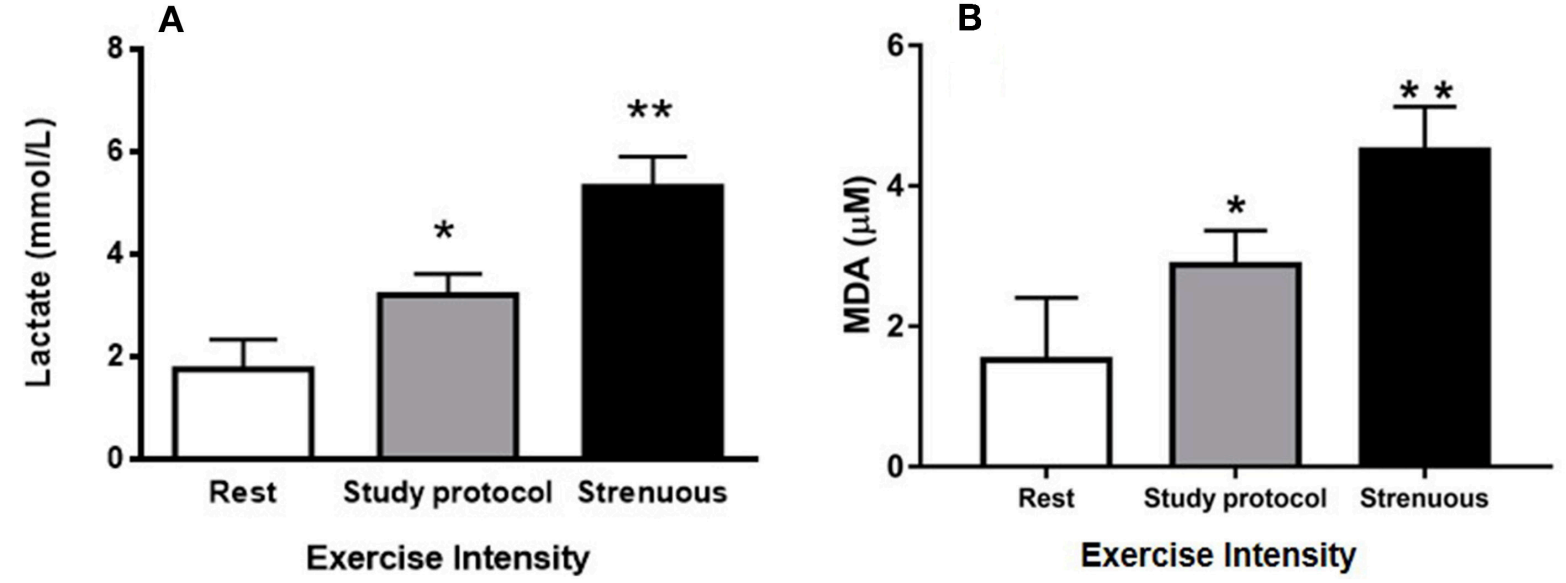
Control of exercise intensity [blood lactate concentrations **(A)** and TBARs **(B)**]: **(A)** Blood lactate concentrations were measured at rest as well as after a session of study protocol and intense exercise. **(B)** MDA concentrations (lipid peroxidation) were measured at rest as well as after a session of study protocol and intense exercise. The animals used in these dosages, lactate (*n* = 15) and lipid peroxidation (*n* = 15), did not participate of the experimental groups. (*P < 0.01 compared to rest, **P < 0.001 compared to rest and study protocol). Values are expressed as mean and ± SD. Data were analyzed by one-way ANOVA followed by Tukey's multiple comparison test.

The blood lactate levels were measured under three different conditions to verify that the study protocol imposed an exercise of moderate intensity; (1) animals at rest, (2) animals immediately after being submitted to the study protocol, and (3) animals immediately after being submitted to a known strenuous exercise protocol. At rest, the blood lactate concentration was 1.78 mmol/L. Following the study protocol, the measured concentraton was 3.23 mmol/L and it was 5.63 mmol/L after a strenuous exercise (Figure 1A). These data suggest that the exercise protocol employed in this study was associated with moderate intensity, since the lactate level of animals submitted to strenuous exercise was significantly greater than level of the study protocol. In addition, there was a change of 88% in lipid peroxidation from the rest group (1.52 μM of MDA) to moderate exercised group (2.87 μM of MDA). However, the strenuous exercised mice (4.50 μM of MDA) presented an increase of 196% in lipid peroxidation when compared to rest group (Figure 1B). These data suggest the animals did not suffer much oxidative stress from our study protocol.

### Effect of Exercise on Development of Murine Leishmaniasis

**Figure 2:**
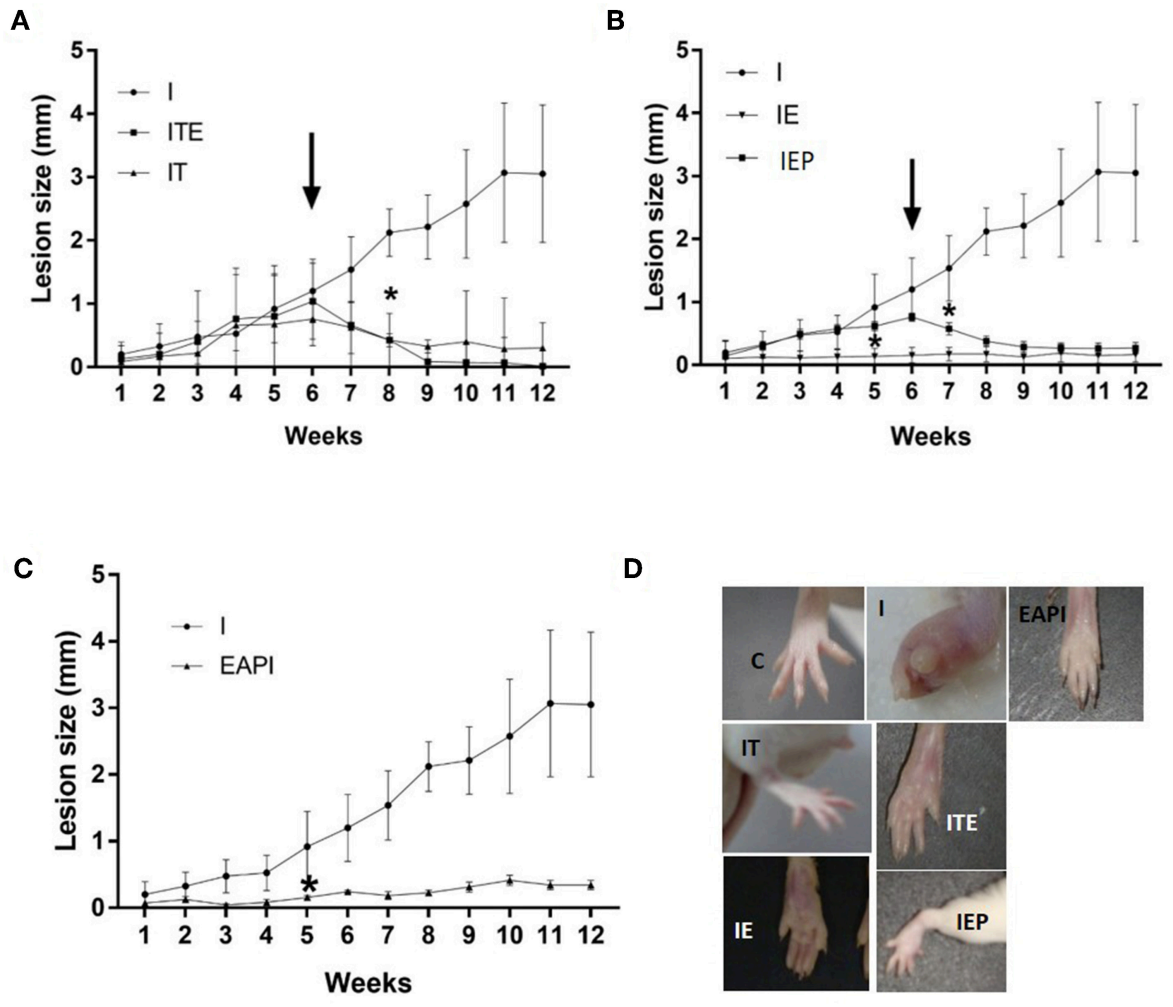
Effect of exercise on the development of lesions on the paws of BALB/c mice that were infected with L. major (2 x 106 promastigotes): **(A–C)** The size of paws was measured weekly for 12 weeks with a pachymeter (Mitutoyo). Results are expressed as the difference between the contralateral paw and infected paw (mm). The arrow indicates the start of training and/or treatment. Values represent the average of two experiments with 8 animals per group in each experiment. **(D)** A representative image of the infected footpad from 1 animal of each different group at the end of the assay (13th week). Values were expressed as mean ± SD. I—Infected. IT—Animals infected and treated with therapeutic dose (8 mg) of Glucantime® after the onset of injury. ITE - Animals infected, trained and treated with therapeutic dose (8 mg) of Glucantime® after the onset of injury. IE—infected animals trained from the first week of infection. IEP—infected animals trained after the onset of injury (6th week). EAPI—Animals trained for 6 weeks, infected and trained for 12 weeks. * The week displaying significant difference (*P* < 0.001) from the control group (I).

After 12 weeks of infection, it was observed that treatment with therapeutic dose of Glucantime® after the appearance of the lesion (IT) led to a regression in the lesion size consistent with inhibiting their progression (Figure 2A). This was considered our experimental control that represented current practice for clinical care. The development of lesion size due to infection in control animals (I) was significant only from the 6th week after injection of the inoculum of *L. major* (*P* < 0.01 compared to the first week of infection). Animals that performed exercise from the beginning of study period when the infection was introduce (IE) displayed a complete inhibition in the progression of the lesion (Figure 2B). Even when exercise was begun at 6 weeks post-infection, when a lesion had been visually established (IEP), a regression was observed in the lesions (Figure 2B). The animals in the ITE group, which were treated with Glucantime® and submitted to the training protocol after the onset of injury, showed the same pattern presented by the IT group (Figure 2A). Interestingly, the prophylaxis group that was subjected to 6 weeks of training before infection (EAPI) did not develop lesion (Figure 2C). These data suggest that exercise may modulate the immune response to leishmaniasis.

### Intradermal Response to Total Leishmania Antigen

**Figure 3:**
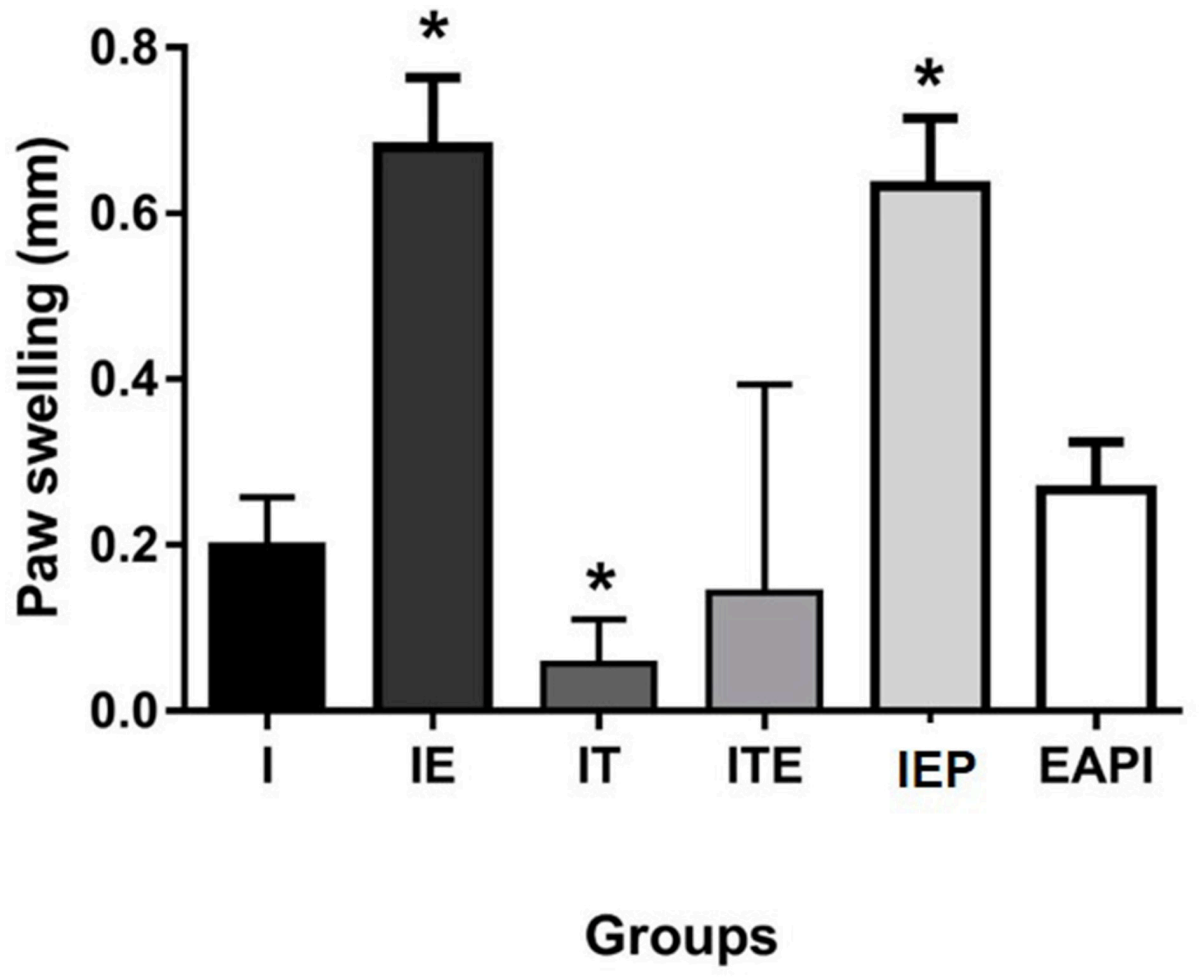
Delayed type hypersensitivity (DTH) to total Leishmania major antigen in BALB / c mice trained and/or treated with Glucantime®. DTH was carried out in the 13th week, at the end of the experiment. The DTH was evaluated using 20 μg of total L. major antigen in 20 μL of saline inoculated into the contralateral non-infected paw. Results obtained after 48 h of antigen inoculum of L. major. Values are expressed as mean ± SD. Data were analyzed by one-way ANOVA followed by Tukey's multiple comparison test. *P < 0.05 compared to (I).

The DTH response was measured 48 h after an inoculum of *L. major* was injected into the uninfected paw (Figure 3). IE and IEP groups had significant differences compared to control infection (I) showing a positive DTH. The group treated with a therapeutic dose of Glucantime® showed no positive DTH as well as EAPI and ITE groups.

#### Parasite Load

**Figure 4:**
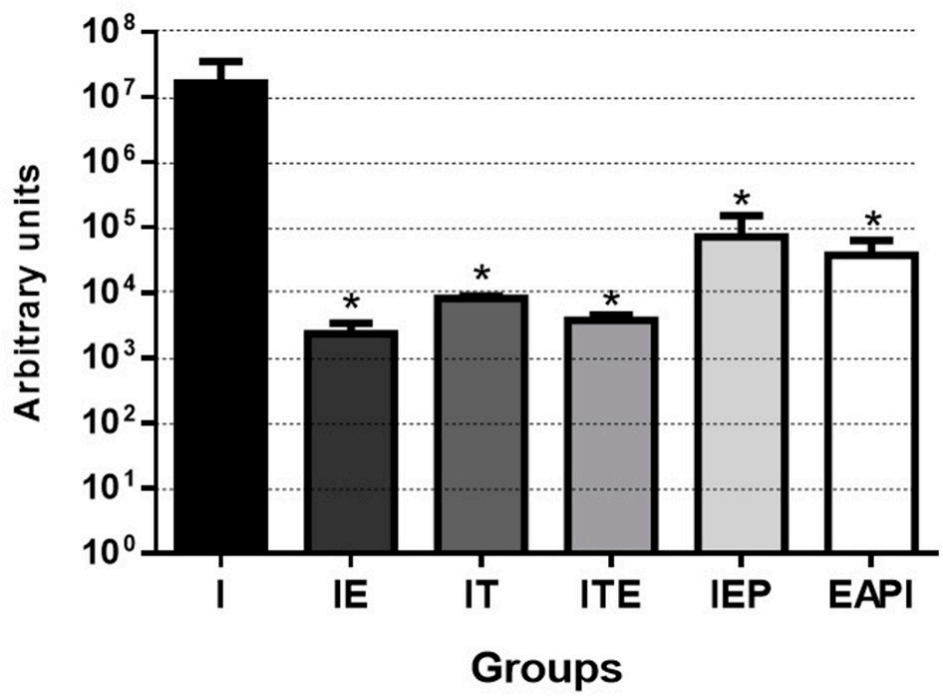
Parasitic burden of infected mice. Parasites were isolated from the paws after 13 weeks of infection and quantified by serial dilution in Schneider's medium plus 20% FBS. After isolation, they were kept at 26°C until the presence of parasites was observed in group I (7th day). Then, the parasites were quantified in Neubauer Chamber, and the number of parasites obtained was divided by the total weight (gram) of infected paws tissue. Values are expressed as the mean of two experiments with the samples of eight animals per group grouped as a pool. The bars represent the standard error of the mean. I—Infected. IT—Animals infected and treated with a therapeutic dose (8 mg) of Glucantime®, after the onset of the lesion. ITE—Animals infected, trained and treated with therapeutic dose (8 mg) of Glucantime® after the onset of injury. IE—Animals infected and trained from the first week of infection. IEP—Animals infected and trained after the appearance of the lesion (6th week). EAPI—Animals trained for 6 weeks, infected and trained for another 12 weeks. *P < 0.05 with respect to I.

We observed that the number of promastigotes grown from the parasites isolated in the infected control group (I) was, at least, 230 times higher than in the other groups, reaching this difference, at 7,000 times when compared with IE group (Figure 4).

### Effect of Exercise on the Production of Cytokines by Infected Animals

#### Cytokine Production in Lymph Node Cells Draining the Lesion

**Figure 5:**
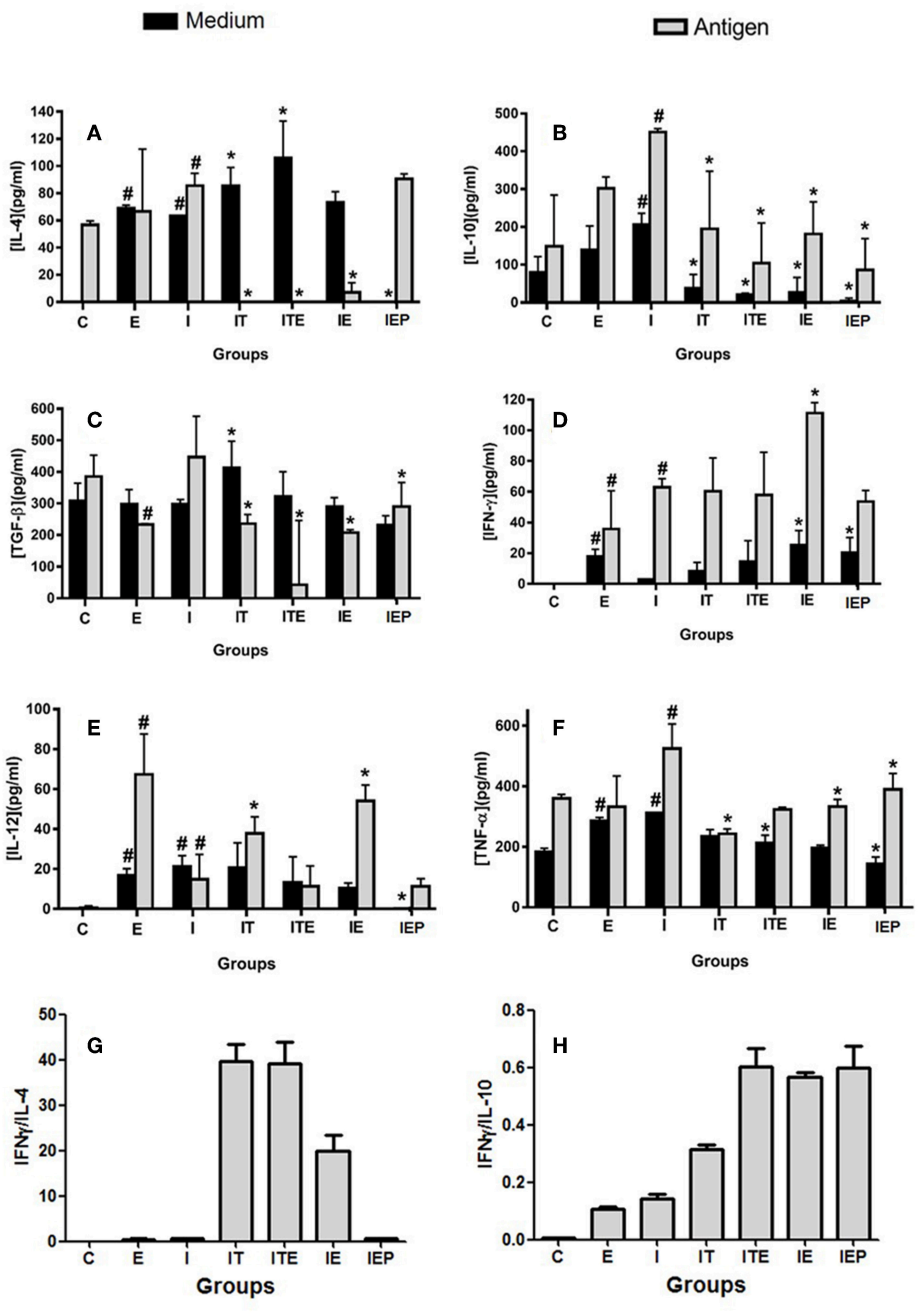
Effect of physical exercise on cytokine production by lymph node cells of BALB/c mice. The concentration of cytokines was determined by ELISA according to Material and Methods. Values represent the mean of two experiments with samples of the eight animals per group grouped as a pool. **(A)** IL-4; **(B)** IL-10; **(C)** TGF-β; **(D)** IFN-γ; **(E)** IL-12; **(F)** TNF-α; **(G)** IFN-γ/IL-4 ratio; **(H)** IFN-γ/IL-10 ratio. The bars represent the standard error. C—Sedentary and uninfected. E—Trained not infected. I—Infected. IT—Animals infected and treated with a therapeutic dose (8 mg) of Glucantime®, after the onset of the lesion. ITE—Animals infected, trained and treated with therapeutic dose (8 mg) of Glucantime® after the onset of injury. IE—Animals infected and trained from the first week of infection. IEP—Animals infected and trained after the appearance of the lesion (6th week). *#P* < 0.05 in relation to C and in *P < 0.05 in relation to I.

The IL-4 cytokine was not detected in the C and IEP, with very similar concentrations in the other groups (Figure 5A). The IE, IT, and ITE groups had IL-4 production inhibited after stimulation with the parasite antigen (Figure 5A), where the other groups showed no significant difference from group I. The production of IL-10 was greatest in the infection control group (I), which was significantly stimulated by the parasite antigen (Figure 5B). Although this profile was also presented by groups E, IE, IT, and ITE, the increase promoted in I was significantly higher (Figure 5B). The production of TGF-β was very similar in all groups that were not stimulated, and was more pronounced in group I after stimulation with the parasite antigen (Figure 5C). The presence of IFN-γ was not detected in the supernatant of both cells, non-stimulated cells and stimulated by the *L. major* antigen in group C (Figure 5D). In groups E and IE, this cytokine was shown to be increased in comparison to its respective C and I controls in both, non-stimulated and stimulated cells (Figure 5D). The cytokine IL-12 was not detected in the both systems non-stimulated and stimulated from C group (Figure 5E). Again, in the E and IE groups, the cytokine in question was shown to be increased in relation to its respective C and I controls in the system stimulated by *L. major* antigen (Figure 5E). The Group I presented the highest TNF production in both systems, where the differences with the other groups, although statistically significant, were not very pronounced (Figure 5F). To compare the cytokines pattern production (Th1 and Th2), we calculated the ratio of IFN-γ/IL-4 and IFN-γ/IL-10. The groups IT, ITE and IE showed a higher ratio for IFN-γ/IL-4 (Figure 5G), while for IFN-γ/IL-10, the largest ratios were observed for the exercised mice (ITE, IE, and IEP) (Figure 5H).

#### Cytokine Production in Infected Paw Cells

**Figure 6:**
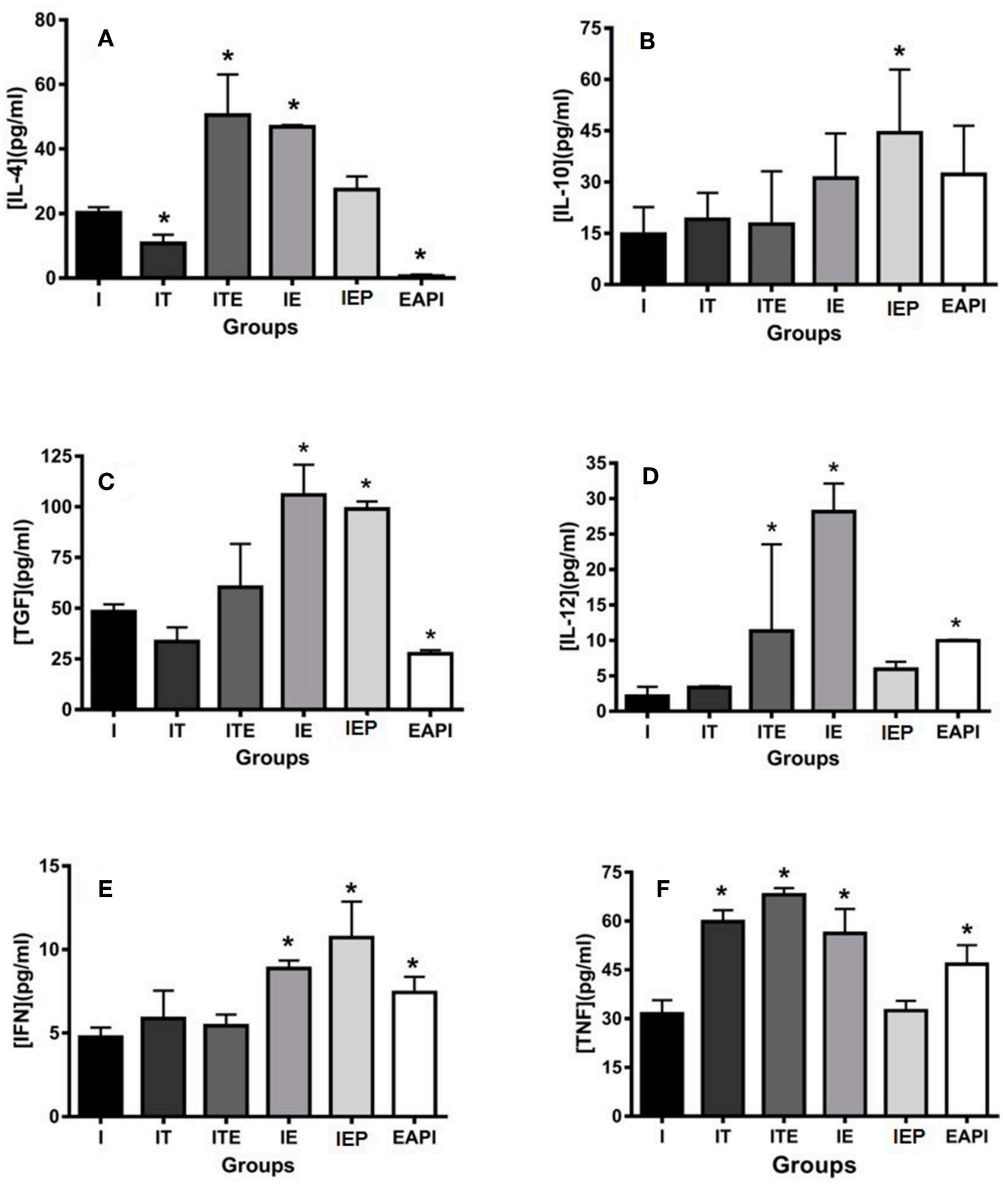
Effect of physical exercise on cytokine production by cells in the paws of infected BALB/c mice. The cytokines IL-4 **(A)**, IL-10 **(B)**, TGF-β **(C)**, IL-12 **(D)**, IFN-γ **(E)** and TNF **(F)** were determined by ELISA (see Material and Methods). Values represent the mean of two experiments with samples of the eight animals per group grouped as a pool. The bars represent the standard error. C—Non-infected sedentary. E—Trained not infected. I—Infected. IT—Animals infected and treated with a therapeutic dose (8 mg) of Glucantime®, after the onset of the lesion. ITE—Animals infected, trained and treated with therapeutic dose (8 mg) of Glucantime® after the onset of injury. IE—Animals infected and trained from the first week of infection. IEP—Animals infected and trained after the appearance of the lesion (6 th week). EAPI—Animals trained for 6 weeks, infected and trained for another 12 weeks. *P < 0.05 with respect to I.

The animals submitted to physical exercise presented an increase in the production of inflammatory cytokines IL-12, IFN-γ, and TNF, with an exception for IEP for IL-12 and TNF and ITE for IFN-γ (Figures 6C–E). Though group I presented a production of inflammatory cytokines lower than the exercised groups, it cannot be said that this group presents an anti-inflammatory profile because the production of these cytokines in the paw of infected animals was higher in the groups submitted to the exercise. The EAPI group showed a more pro-inflammatory pattern, with the concentrations of the anti-inflammatory cytokines IL-4 (Figure 6A) and TGF-β (Figure 6C) lesser than I and the regulatory cytokine IL-10 (Figure 6B) higher than I.

## Discussion

The potential benefits promoted by physical exercise with regards to immune response appears to depend on the level of exertion experienced by the individual. In practice, the absence of training control can lead to overtraining such as in the case of strenuous training that may not promote the beneficials adaptations (Kellman, 2010; Walsh et al., 2011a). As a consequence, there is a possibility of an increase in viral infections or intracellular microorganisms, since high intensity exercises direct the immune system to a predominance of humoral response (Th2), which is not able to overcome these types of infection. In contrast, the practice of controlled exercise to provide a moderate physical intensity, results in a predominance of the cellular response pattern (Th1), controlling these kind of infections (Walsh et al., 2011b).

There are several parameters currently used to control exercise volume and intensity. The gold standard is represented by the maximum oxygen consumption (VO_2_max). However, heart rate, subjective perception of effort, blood lactate concentration and specific physical capacity tests can also be employed (ACSM, 2011). Here, the blood lactate concentration in mice submitted to a session of exercise (moderate or strenuous) were evaluated to verify that the study protocol reflected a moderate exercise. In the post-exercise period of study protocol (3.23 mmol/L), the lactate level was characteristic of a moderate intensity exercise, since the concentration for a protocol of intense exercise until fatigue (5.63 mmol/L) was significantly larger (Figure 1A). Other protocols of moderate intensity presented lactate values very similar to those found in this work. Balb/c mice submitted to a moderately intensive run (25 m/min) presented a blood lactate concentration of 2.61 mmol/L, whereas at rest the value found was 1.81 mmol/L (Haramizu et al., 2009). This value is similar to the value measured in this work (1.78 mmol/L at rest). In addition, the mice submitted to a strenuous exercise presented a lipid peroxidation level that was 196% greater than the rest group, while the study protocol group increased 88% (Figure 1B). This is a further indication that the intensity of the protocol used was moderate, since the production of reactive oxygen species (ROS) by the muscle is directly proportional to the increase in exercise intensity (Bloomer and Fisher-Wellman, 2008). The levels of lipid peroxidation are increased after exhaustive aerobic exercise and resistance exercise (Alessio et al., 2000; Pinho et al., 2010).

Additional factors associated to increases in lipid peroxidation include the physical fitness level and the antioxidant capacity (Pinho et al., 2010). The training protocol implemented in this study included an adjustment to the overload weight applied to the animals during swimming every four weeks to maintain the desired intensity. This load adjustment is necessary for two reasons: (1) the timeframe of the experiments was during a growth phase of the animals when their weight increased and the extra loads are relative to body weight; and (2) the animals adapt to exercise through improved fitness. Thus, a greater stressor stimulus was required for homeostasis breakdown. These changes reflected an adaptation of the animals to the exercise, which was confirmed by the increased fitness level of the animals (Table 1). These adaptations are generally associated with increases in antioxidant capacity. Since the maintenance of the intensity and volume parameters of an exercise is essential to achieve the adaptations promoted by it (Walsh et al., 2011a) and the official positioning of the International Society for Exercise and Immunology (ISEI) establishes that a program of physical exercises of mild/moderate intensity improves immune function (Walsh et al., 2011b), the parameters of the swimming protocol executed by the trained animals provided a moderate exertion level that positively modified their immune response to an infection with *Leishmania*. This is consistent with multiple studies on exercise that show, when prescribed according to ACSM recommendations, reduces episodes of infection (Karper and Boschen, 1993; Nieman et al., 1993; Rowbottom and Green, 2000).

Our physical training protocol was able to not only able to avoid the development of lesions (EAPI and IE—Figure 2), it also appeared to reverse an established infection (IEP and ITE—Figure 2) along with a reduction in parasitic burden (Figure 4). Mice trained after a lesion was established displayed a disease progression that was similar to animals treated with Glucantime® over the same period. The delayed hypersensitivity response (DTH) in the trained Balb/c mice (IE and IEP) was significantly higher in relation to the infected group (I) (Figure 3). The positive DTH is indicative of a cellular immune response (Th1), which appears generally to correlate with protection against parasites of the genus *Leishmania* (Naderer and Mcconville, 2008; Mougneau et al., 2011). Although the infection was also contained in the IT and ITE groups, the DTH appeared equal to or less than group I (Figure 3). The IT and ITE groups were treated with the reference drug Glucantime®, therefore the direct antiparasite effect of the drug induced control of infection. However, the literature is not clear on this issue and the mechanism of action of pentavalent antimonials has not yet been fully elucidated (Baiocco et al., 2009; Kaur and Rajput, 2014; Brazilian Ministry of Health, 2017). Likewise, the correlation between a therapeutic response and immunity is unclear (Conceição-Silva et al., 2018). Similar findings have been achieved in *L.major*-infected mice after immunization with lpg2- *L. major* (Uzonna et al., 2004) and Lmp27^−/−^ mutant (Elikaee et al., 2018), where protection and DTH are not necessarily correlated.

The infected group (I) had a high prevalence of parasites in the infected paw how can be observed in the estimate of parasite load (Figure 4). Animals that underwent the moderate exercise protocol at the time of lesion appearance presented an estimate parasite load nearly 300-fold lower than that of infected animals (IEP and EAPI—Figure 4) or 4,000 fold lower in the case of ITE. Animals that began the exercises at the time of the infection, the difference was 7,000 fold (IE—Figure 4). The estimate parasite load appeared to be inversely proportional to DTH, except for the animals treated with the drug (IT—Figures 3, 4). In a previous study on the development of leishmaniotic lesions caused by *L. major* (2 × 10^6^ promastigotes) injections in susceptible Balb/c and resistant C57BL/6 mice (Barthelmann et al., 2011), the course of the lesion size, DTH and parasite in resistant animals presented positive DTH and low parasite load similar to that found in this study for susceptible mice submitted to exercise. These observations corroborate our hypothesis that moderate exercise can promote a protective and curative effect against *Leishmania*. Although we have not evaluated the parasitic load in the draining lymph nodes, it is known that even in cured animals the persistence of parasites in the draining lymph node occurs for long periods. The persistence of parasites seems to be important for the maintenance of the cellular immune response and generation of T cell memory (Mandell and Beverley, 2016; Conceição-Silva et al., 2018).

During a Th1 response, the increased production of IFN-γ stimulates the expression of the enzyme iNOS resulting in the production of nitric oxide (NO) by macrophages. The expression of this enzyme is induced by various stimuli, including IL-1, TNF, IFN-γ, and LPS (Stafford et al., 2002; Mansueto et al., 2007). The NO appears to be the main molecule involved in leishmanicidal mechanisms (Liew et al., 1990). In an anterior study from our group using the same exercise protocol, macrophages from 12 weeks trained Balb/c mice showed a significant increase in NO production after LPS stimulation (Terra et al., 2013). Here, mice infected and submitted to physical exercise (IE) had significantly higher concentrations of Th1 cytokines than the infected sedentary (I) in the isolated cells of the popliteal lymph node. This pattern was also observed for the uninfected animals submitted to exercise (E) when compared to the control system (C) (Figure 5). There was a significant decrease in IL-10 production by lymphocytes isolated from trained animals (Figure 5B), mainly in animals that were trained from the 6th week of infection (IEP).

Qualitatively, the production of these cytokines can be compared to the Th1/Th2 dichotomy models afforded by the resistant C57BL/6 mouse (Fritzche et al., 2010) and the susceptible Balb/c mouse (Barthelmann et al., 2011). In Balb/c mice, IL-10 is associated with the phenomena of susceptibility to infection by intracellular microorganisms, such as *Leishmania major* (Sacks and Noben-Trauth, 2002). The cytokine IL-10 was initially described as a Th2-type cytokine (Belkaid, 2007). Additional evidence has shown that its production is associated with regulatory T cell response (Treg) (Vignali et al., 2008). However, IL-10 production is not Th2 or Treg specific. It can also be expressed by other cells including Th1, Th17 subgroups, CD8^+^ cells and B lymphocytes (Maloy and Maloy and Powrie, 2001; Roncarolo et al., 2006; O'Garra and Vieira, 2007; Trinchieri, 2007; Saraiva and O'Garra, 2010). Further, IL-10 can also be produced by cells participating in the innate immune response such as dendritic cells (DCs), macrophages, mast cells, NK cells, eosinophils and neutrophils (Saraiva and O'Garra, 2010). The decrease in IL-10 and TGF-β measured in the IEP group may play an important role in the resolution of the infection since the inflammatory cytokines IFN-γ and IL-12 were not modified in this group at the end of 12 weeks of infection in the systems stimulated by the parasitic antigen (popliteal lymph node) (Figures 5D,E). This was significantly different from that presented by the group that was trained from the first week of infection (IE), where the decrease in IL-10 and TGF-β (Figures 5B,C), production and the increase in IFN-γ and IL-12 production occurred concurrently (Figures 5D,E). It is possible that this difference may be due to the shorter training time to which this group (IEP) was submitted (6 weeks only). However, this hypothesis can be confirmed only if the IE cytokine training pattern is analyzed in the 6th week. Although TNF is an inflammatory cytokine (Mougneau et al., 2011), it has been decreased in the systems of mice submitted to exercise (IE and IEP) compared to infected animals (I) (Figure 5F). Our findings are in agreement with the literature where it has been widely argued that the practice of moderate-intensity physical exercise leads to a predominance of Th1 cytokines (Pedersen and Hoffman-Goetz, 2000; Walsh et al., 2011b).

Although the levels of IFN-γ detected of the trained (ITE, IEP)/treated (IT) groups were relatively similar to infected group (I), the levels of anti-inflammatory cytokines as IL-4, IL-10, and TGF-β were significantly lower. We hypothesize that in the absence of a strong IL-4 or IL-10 response, the low levels of IFN-γ produced may be sufficient for protection. When we performed the IFN-γ/IL-4 and IFN-γ/IL10 ratio, we observed a clear slope for the Th1 response (Figures 5G,H). These data are consistent with the cytokine patterns of the lymph node cells after 12 weeks training of Balb/c mice, when compared with sedentary group (Terra et al., 2013).

A similar scheme can be observed at the site of infection. When we analyzed the cytokine profile produced in the infected paw (Figure 6), we observed that the response tended to be of a Th1 type where cytokines associated with this pattern (IL-12 and IFN-γ) are increased in the three of the four exercised groups studied including IEP. The local increase in this group could possibly contribute to the resolution of the infection. The presented EAPI cytokine profile at the site of infection seems characteristically to trend toward the Th1 pattern. The inflammatory cytokines IL-12 and IFN-γ, as well as TNF, were found to be increased (Figures 6D–F), whereas those associated with the Th2 pattern were significantly decreased (IL-4 and TGF-β) (Figures 6A,C) or without significant difference (IL-10) (Figure 6B). One possibility for this well-established pattern could be associated to training time, since this group began the practice of physical exercise at 6 weeks before the infection, and maintained their training throughout the course of the experiments. Data from the EAPI group suggest that regular physical exercise of moderate intensity can modulate the Th1 response and provide protection against *L. major* infection.

A regulatory response also appears to be present, especially in the IEP group, where IL-10 and TGF-β cytokines have been found to be increased (Figures 6B,C). The phenomena of resistance and susceptibility in all forms of leishmaniasis have been related to immune responses mediated by cells (Belosevic et al., 1989). Individuals with cutaneous leishmaniasis in the New World present a positive prognosis when their cellular immune response is balanced (Brazilian Ministry of Health, 2017). On the other hand, susceptibility is associated with an activation response of Th2-type lymphocytes (T helper 2), with production of interleukin 4 (Bogdan et al., 1996; Romagnani and Abbas, 1996; Lanouis et al., 2002; Mansueto et al., 2007). Diffuse cutaneous leishmaniasis and visceral leishmaniasis present non-protective Th2 responses (Romagnani and Abbas, 1996; Mansueto et al., 2007). However, an exacerbation of the Th1 type response (hypererygia) leads to increased tissue destruction where parasite antigen is present. This is characteristic of classical mucosal leishmaniasis, where IFN-γ and TNF levels are very high, associated with a relatively low production of IL-10. In addition, cells from individuals with this parasite have low ability to respond to cytokines inhibiting IFN-γ secretion (Brazilian Ministry of Health, 2017).

Overall, our data clearly shows a protective effect from moderate exercise on the development of experimental leishmaniasis. A potential mechanism for this protection is a modulation in the cytokine pattern during the immune response that was promoted by exercise. Further studies are needed to elucidate additional details in these complex, interconnected mechanisms.

## Ethics Statement

This study was approved by the Ethics Committee for Experimental Use and Animal Care at the Biology Institute Roberto Alcântara Gomes (UERJ) (Protocol number CEUA/019/2015).

## Author Contributions

RT conducted experiments and contributed to the writing of the manuscript. PA, AL and SG, conducted the experiments. LR contributed to the execution and discussion of cytokine production analysis. VS conducted co-orientation and contributed to the writing of the manuscript. SD-S conducted experiments, co-orientation and contributed to the writing of the manuscript. PD conducted orientation, experimentation and contributed to the writing of the manuscript.

### Conflict of Interest Statement

The authors declare that the research was conducted in the absence of any commercial or financial relationships that could be construed as a potential conflict of interest.
